# Religious Participation, Gender Differences, and Cognitive Impairment among the Oldest-Old in China

**DOI:** 10.4061/2010/160294

**Published:** 2010-06-17

**Authors:** Wei Zhang

**Affiliations:** Sociology Department, University of Hawaii at Manoa, 2424 Maile Way, Saunders 204, Honolulu, HI 96822, USA

## Abstract

This study examines if religious participation in China is associated with cognitive functioning among the oldest-old and whether positive psychological feelings and leisure activity engagement explain the association, and gender moderates the association. Logistic regressions were used to analyze the Chinese Healthy Longevity Survey. A significant negative association between religious participation and cognitive impairment was found among the oldest-old and much of the association was mediated by positive psychological feelings and leisure activities. Women reported higher proportion of religious participation, but the cognitive benefits of religious participation were stronger for men. Findings indicate that (a) religious participation is significantly correlated with cognitive functioning in part because the religious oldest-old are more likely to be optimistic and happy and engage in more cognitively stimulating activities; (b) there might be gender differences in religious participation such that the oldest-old men may engage in religious activities that are particularly relevant to cognitive functioning.

## 1. Introduction

Despite the recent advances in research on religion and health in general (for reviews, see [1, 2]), only a few studies have explored the relationship between individual religious involvement and cognitive functioning among older adults [3–6]. Virtually no studies have examined this relationship in China. This study intends to fill this gap in the literature by examining whether religious participation is associated with cognitive functioning among the oldest-old (80 years and older) Chinese, if it is, what mediates the association, and whether gender conditions the association. 

The examination of religion and health association in China has increasingly become an urgent priority. China, the most populous country in the world, is now aging at a rapid speed. According to Zeng et al. [7], the number of the oldest-old population, currently the largest of any nation in the world, may increase by more than 100 million by 2050. Such rapid growth of the oldest-old predicts a future burden of cognitive and functioning disability [8]. Therefore, it is increasingly critical to study social factors such as religious participation that might be beneficial to the maintenance of cognitive functioning of older Chinese, especially considering the decline of the younger population due to the “one-child policy” and the possible lack of family and societal care of older adults in China. 

In the following theoretical background section, psychosocial explanations of why religious participation is related to cognitive functioning are summarized first. Then, the cognitive functions of Chinese religions are proposed and the conceptual framework that guides this study is outlined. Finally, gender differences in religious participation as well as the impact of religious participation on cognitive functioning are discussed.

## 2. Theoretical and Empirical Background

### 2.1. Previous Research on Religion and Cognition Functioning

Although religion and health association is well documented (e.g., [1, 2]), only a few studies have explicitly looked at religion in its relation to cognitive functioning and dementia in late life [4, 6]. Van Ness and Kasl [6] were among the first to examine the effects of religious attendance and religious identity on cognitive functioning. Using a sample of 2,812 men and women aged 65 and over residing in New Haven and controlling for a wide range of confounders, they found that religious attendance, rather than religious identity, was associated with a reduction in the odds of cognitive dysfunction over 6 years. Hill and colleagues [4] investigated whether religious attendance was associated with slower rates of cognitive decline. Using a four-wave data set collected from a sample of 3,050 older Mexican-origin individuals, they constructed a series of linear growth curve models and demonstrated that religious attendance was associated with slower rates of cognitive decline among older Mexican Americans over 8 years. 

Several lines of theories have been put forth explaining why religious involvement might be associated with healthy cognitive aging. First, religious involvement is related to a greater sense of coherence, meaning, and hope, which may help individuals cope effectively with increasing stress, anxiety, and depression associated with advanced age [9]. For instance, various aspects of religious involvement including sermons, prayer, scriptural reading, singing, and philosophical discussions may directly or indirectly buffer against cognitive decline through enhancing positive psychological feelings such as optimism and happiness. 

In addition, religious involvement, as an important form of active lifestyle and social engagement, may help to develop other forms of leisure activities that require the mobilizations of cognitive faculties, which augment brain reserve capacity [10, 11], thus delay the deterioration of cognitive ability. According to Bassuk and colleagues [3], social engagement, defined as the maintenance of rich social connections and a high level of participation in social activities, not only provides dynamic environment that has high cognitive demands, but also indicates a commitment to community and engenders a health-promoting sense of purpose and fulfillment. A population-based longitudinal study conducted by Newson and Kemps [12] suggested that after controlling for sensory functioning, activity was a significant predictor of current levels of speed, picturing names, incidental recall, and verbal fluency, and other dimensions of cognitive ability. There is also epidemiological evidence supporting that a lifestyle characterized by engagement in leisure activities of intellectual and social nature was associated with slower cognitive decline for healthy older adults and may reduce the incidence of dementia [13].

### 2.2. Chinese Religions and Cognition

Different from the congregational religious traditions in Western society where many—perhaps most—studies in the area of religion and health have gauged religious involvement in terms of affiliation and/or self-reported religious behavior, such as the frequency of attendance at services [14], religious participation in China is not only a unique form of social engagement, but also a unique form of lifestyle and life philosophy. Various forms of Chinese religions have long-standing and distinct psychosocial and behavioral functions on health and longevity (e.g., [15–17]. Most people in China practice multiple religious traditions such as folk religion, Confucianism, Taoism, and Buddhism. 

For instance, followers of Confucian teachings believe that virtues such as self-cultivations, chung (loyalty), shu (empathy), and jen (humaneness) are imperative to develop and maintain deep and profound secular relationships with self, family, community, and state [18]. These communication skills may greatly facilitate complex interpersonal exchanges and help to develop rich social networks, on the basis of which coreligionists are likely to engage in various leisure activities that are socially integrated or cognitively stimulating such as collective exercises, reading, playing cards, chase, or Mah-jong [17, 19]. In this paper, engaging in various leisure activities is considered as one of the important mediators linking religious participation and cognitive functioning among older Chinese. 

Buddhism is more similar to Western religions with respect to spiritual and ritual practices, which are likely to offer wisdom to one's ultimate concerns about the meaning of life and provide means to reach wholeness, transcendence, or enlightenment. The practice of Buddhism may help in achieving spiritual well-being characterized by tolerance, a sense of harmony, inner freedom, and peace [15]. These faith-based attitudes, along with ritual practices such as prayer and temple visiting, may enhance feelings of confidence and a sense of personal control and lead to positive expectations such as hope, optimism, and happiness [20, 21]. Optimism may not only promote longevity, physical well-being, and health-promoting behaviors [20, 22–25], but also help in coping effectively with various traumatic life events associated with aging. In this study, positive psychological feelings such as optimism and happiness are considered another set of mediators linking the association between religion and cognitive functioning. 

Among different religious traditions, Taoism, indigenous to China, is believed to be the most influential intellectual tradition underlying the development of traditional Chinese medicine and is most relevant to health and well-being. For example, Taoism emphasizes holism: all things are considered interdependent and interactive. Guided by this principle, health phenomena are viewed in light of a web of qi and the holistic well-being can be promoted through energy-based health practices and healing processes such as Qigong [16]. Through deep breathing, concentration, and relaxation, body, mind, and spirit are effectively connected. As one example of Qigong, T'ai Chi'i is relatively easier to learn, thus becomes one of the most popular exercises among older Chinese. 

In sum, Chinese religions have a potential impact on the health and well-being of older adults. They integrate essences from different religious traditions, build social as well as spiritual relationships, encourage an engaged and active lifestyle, and create positive expectations. In this sense, China is a nation of religion, not by a Western definition, but in its own way. Many aspects of Chinese religions are likely to be associated with cognitive functioning. Collectively, the first two hypotheses of this study are presented as the following:

**Hypothesis 1**

Religious participation has an inverse association with cognitive impairment among the oldest-old Chinese.

**Hypothesis 2**

The effects of religious participation on cognitive impairment are likely to be partially mediated by positive psychological feelings such as optimism and happiness, and engagement in a variety of leisure activities.

### 2.3. Religion, Gender, and Cognition

The perception of the importance of religion may vary by social groups such as gender. In China, significant differences in gender roles, opportunities, and obligations disadvantage women, especially the oldest women, in many ways [8]. With respect to cognitive functioning, the incidence and severity of dementia is significantly greater among women in China than men [8, 26–28]. Religious participation, with its possible function of developing social resources and mobilizing cognitive faculties, may be particularly popular among older Chinese women. Accordingly, the third hypothesis of this study is introduced as the following:

**Hypothesis 3**

Gender differentials in religious involvement can be expected such that the oldest-old women tend to report higher proportion of religious participation than the oldest-old men in China.

Oldest-old women may report more religious involvement on average, but will they enjoy more cognitive benefits from the participation than their male counterparts? Prior research suggests two ways to think about gender differentials in the impact of religion on health. First, it is reasonable to argue that women are benefited more than men. For instance, Strawbridge and colleagues [29] found that the beneficial effects of church attendance on mortality are more evident among women than men. They speculated that these gender differences in mortality may be attributed, in part, to the fact that women are more deeply involved in church-based social support systems than men. Additional support for this view was provided by Idler [30], whose study suggested that the more a woman attends religious services, and the better she knows other members of the congregation, the less likely she is to be physically disabled or depressed. In a similar vein, a recent study [17] in China indicated that religious participation, associated with other socially integrated leisure activities, predicts lower mortality risk among oldest-old women, but not among oldest-old men. 

Alternatively, it is also possible to propose that health-protective effects of religion will be more evident among men. For instance, studies in the U.S. suggested that women may face unique challenges in the religious setting that might offset the beneficial effects of church-based support. For instance, research reviewed by Heyer-Gray [31] shows that women are more likely than men to do cleaning and cooking in the church, but less likely than men to lead or say prayers during worship services and to assist in serving communion or reading to the congregation from the Bible. That is, occupying the subordinate position in the church and engaging in nonintellectual tasks may be a source of distress for women, which tends to negate the potential benefits of strong church-based ties. Using data from a longitudinal nationwide survey of members of the Presbyterian Church (USA), Krause and colleagues [32] revealed that even though women receive more emotional support from coreligionists than men, it is men who are likely to enjoy the salutary effects of church-based support on health. Taken together, previous studies suggested potential gender differences in the impact of religion on health, but which gender group gains more remains controversial. Accordingly, the fourth hypothesis of this study is presented as the following:

**Hypothesis 4**

Religious participation is negatively associated with cognitive impairment, and the association is more for one gender group than for the other. This study will examine whether oldest-old women or oldest-old men are more likely to enjoy more cognitive benefits from religious participation in China.

## 3. Methods

### 3.1. Sample

Data to test these hypotheses come from the 1998 Chinese Healthy Longevity Survey (CHLS). There are 9,093 respondents aged 77–122. This survey was conducted in a randomly-selected half of the counties and cities of the 22 provinces where Han Chinese are the majority. These provinces and municipalities are Liaoning, Jilin, Heilongjiang, Hebei, Beijing, Tianjing, Shanxi, Shaanxi, Shanghai, Jiangsu, Zhejiang, Anhui, Fujian, Jiangxi, Shandong, Henan, Hubei, Hunan, Guangdong, Guangxi, Sichuan, and Chongqing, the total population of which is over 980 million, approximately 85.3% of the total population of China. Males and persons over than 90 were oversampled. The overall response rate was 88%. Response rates for variables in the analysis were also high: there are no missing data for age and urban residence, and less than 1% of the data were missing for education, gender, marital status, ethnicity, cognitive impairment, religious participation, activities of daily living, leisure activities, and positive psychological feelings. See Zeng et al. [33] and Zeng and Vaupel [28] for details about the data and its quality, based on which they state that “a careful data quality evaluation (such as reliability coefficient, factor analysis, rates of logically inconsistent answers, etc.) has shown that the quality of the survey is generally good” ([33]). This study focuses on 8,805 respondents aged 80 to 105. Those aged 106 and older were excluded due to insufficient information to validate their old age [28]. After listwise deletion of missing values, the analytical sample was reduced to 8,703.

### 3.2. Measurement

#### 3.2.1. Cognitive Impairment

Cognitive functioning was measured by using the Chinese version of Mini-Mental State Examination (MMSE), which tests four aspects of cognitive functioning: orientation, calculation, recall, and language [34]. The Chinese version of MMSE was appropriately translated from the international standard of the MMSE questionnaire and adopted the necessary changes to make the questions understandable and answerable among ordinary Chinese oldest old, the majority of whom are illiterate [28]. Respondents were asked by 5 orientation-related questions (naming the current time, animal year, season, festival, and county), one naming food question, 6 word recall questions (3 words are mentioned and respondents are asked to repeat them two times), 5 calculations questions (respondents are asked to subtract 3 from 20, then 3 from the previous resulting, and so on), 3 language questions (repeating a sentence and naming simple items such as pen and watch that are shown to the respondents), 1 drawing question, and 3 comprehension questions (respondents are asked to take paper in their right hand, fold it, and then put it on the floor). Responses to the questions were categorized into “incorrect answer” as 0 and “correct answer” as 1. Consistent with previous studies, responses of “unable to answer” were coded as incorrect answers [8, 35]. The range of scores for the MMSE is 0 to 30, with higher scores reflecting better cognitive ability. As has been recommended in the literature, respondents were defined as having “moderate to severe cognitive impairment” (hereafter “cognitive impairment”) if their score of MMSE was less than 18 [8, 36–38]. Using this conventional threshold is to reduce MMSE measurement error because individuals scored less than 18 were likely to be cognitively impaired regardless of education [8, 26, 34]. Approximately 26% of the participants reported having cognitive impairment.

#### 3.2.2. Religious Participation

Religious participation provides substantial compatibility to possibly cover both public and private religious activities, so it may be the most relevant indicator of individual religious involvement while other religious measures are not available. Respondents were asked how often they participated in any kind of religious activities (1 = everyday, 2 = sometimes, 3 = never). A dichotomous variable—religious participation—was created to contrast the everyday and sometimes participants to never participants. More than 16% of the sample reported participating in religious activities at least sometimes.

#### 3.2.3. Activities of Daily Living (ADLs)

Research in the U.S. showed that elderly individuals may attend religious services only occasionally or not at all as a result of functional limitations [4, 39]. ADL limitations also tend to be associated with higher prevalence rates of cognitive impairment [36, 38, 40] and high incidence rates of cognitive decline [41]. So in this paper, ADLs were controlled in the subsequent analyses as suggested by previous studies (e.g., [4, 8]). Respondents were asked to indicate whether they could do any of the following activities entirely by themselves: (a) eating, (b) dressing, (c) transferring, (d) using toilet, (e) bathing, and (f) continence. Responses were coded “without assistance” as (0) and “with any assistance” as (1). A summated index of ADLs, ranging from 0 to 6, was created by counting the number of ADLs that a respondent could not perform independently. The alpha reliability is over  .87, and the mean is 1.05, with a standard deviation of 1.75.

#### 3.2.4. Demographics

Demographic measures include age (in years), ethnicity (Han: 0 = minorities, 1 = Han), gender (female: 0 = male. 1 = female), martial status (married: 0 = not married, 1 = married), education (literature: 0 = illiterate, 1 = literate), and urban/rural residence (urban: 0 = rural residence, 1 = urban residence). The average age of this sample was approximately 92. A majority of the sample was Han (93%), female (60%), rural (62%), illiterate (67%), and unmarried (84%).

#### 3.2.5. Positive Psychological Feelings

Positive psychological feelings were measured by optimism and happiness. Respondents were asked how often they (a) looked at the bright side of the things and (b) felt happy. Responses to these two items were coded 1 (never), 2 (seldom), 3 (sometimes), 4 (often), 5(always), or 8 (not able to answer). A dichotomous variable—optimism—was created by contrasting those whose answers were “often” and “always” to those whose answers were otherwise. Happiness was coded in the same manner. Over 70% and 44% of the respondents reported high levels of optimism and happiness, respectively.

#### 3.2.6. Leisure Activities

Engagement in leisure activities includes exercising, garden work/growing vegetables, reading newspapers or books, playing cards or mah-jong, watching TV, or listening to the radio, some of which have been used in previous studies (e.g., [6]), and some of which were highly relevant in the Chinese cultural setting [17, 19]. All were measured as dummy variables to contrast sometimes or more participants with never participants.

### 3.3. Analysis

The characteristics of the Chinese oldest-old by gender were examined first. Then, a series of nested logistic regression models were examined to estimate how the odds of having cognitive impairment are associated with religious participation, control, meditating, and moderating factors. Model 1 tests the association between religious participation and cognitive impairment, adjusting for demographics and ADLs. In models 2-3, the proposed mediators were added to see how much they could account for the focal association. Moderating effects of gender were examined by creating a cross-product terms between religion and gender, which was entered into the final main effects model (Model 3). To provide insights into the moderating effects of gender, split-sample regressions (separate for men and women) were then analyzed. For each subgroup, the same modeling strategies were applied.

## 4. Results

The descriptive statistics in Table 1 demonstrate substantial gender differences in a variety of characteristics. As expected, oldest-old Chinese women were older, less educated, more likely to be single, and report more ADL problems in general. For instance, more than 87% of oldest-old women were illiterate, compared with 37% of oldest-old men. Only 5% of oldest-old women were currently married compared with over 33% of their male counterparts. In addition, oldest-old women were much less likely than oldest-old men to participate in all five leisure activities and to be optimistic and happy. The prevalence of cognitive impairment for oldest-old women was approximately 2.5 times as high as that for oldest-old men. However, in terms of religious participation, oldest-old women were two times more likely than oldest-old men to participate in religious activities. Therefore, data supported gender differences in religious participation such that oldest-old women reported more religious involvement than oldest-old men in China.

### 4.1. Religious Participation, Mediators, and Cognitive Impairment

Logistic regression models were then applied to estimate the prevalence of cognitive impairment relative to religious participation, control variables (e.g., age, education, urban/rural residence, marital status, and ethnicity), mediating variables (e.g., positive psychological feelings and leisure activities), and moderating factor (e.g., gender). Model 1 tests the association between religious participation and cognitive impairment, adjusting for demographics and ADLs. In models 2-3, psychosocial mediators were added to see how much they may account for this association. 

When the whole sample (*N* = 8,703) was examined (not shown), the odds ratio (OR) in the first model suggested that religious participation was significantly related to lower odds of cognitive impairment (OR = .52, *P* < .001) with adjustment for age, gender, education, urban/rural residence, marital status, and ethnicity. Indicators of positive psychological feelings were added in Model 2 and both were significant. The unstandardized regression coefficients of religious participation reduced from −.66 (in Model 1) to −.55 (in Model 2), indicating that measures of positive feelings account for some of the differences of religious participation on cognitive functioning. Model 3 includes leisure activities, which explained another 22% of the effects of religious participation. Adding the gender and religion interaction term into the final full model (Model 3) suggests that the interaction effects for gender (OR = 1.60, *P* = .07) were moderately significant over and above all the controls and mediators.

### 4.2. The Interaction of Religious Participation and Gender

To provide more insight into the moderating effects of gender, the sample was divided into two subgroups: men and women. For each subgroup, the same modeling strategies were applied.  Table 2 presents the odds ratios and unstandardized coefficients from the logistic regression models for women. Results show that participating in religious activities was significantly associated with lower odds of cognitive impairment after adjusting for demographic factors and ADLs for women (OR = .56, *P* < .001). The regression coefficients of religious participation reduced from −.58 (in Model 1) to −.47 (in Model 2) and then to −.36 (in Model 3), suggesting that indicators of positive psychological feelings and engaging in leisure activities such as exercises, playing cards/mah-jong, and watching TV partially mediated the effects of religious participation on cognitive impairment. Despite of the reduction in the coefficients, however, the effects of religious participation remained significant at.01 levels in Model 3, which indicates its direct and sizeable association with cognitive functioning for women. 

Consistent with previous findings about correlates of cognitive impairment [8], results in Model 1 of Table 2 also showed that age and ADLs were positively associated with cognitive impairment, whereas being literate or married, and living in urban areas were negatively related to cognitive impairment. For instance, each year increase in age changes the odds of cognitive impairment by 11%, and each unit increase in the ADLs results in an increase of cognitive impairment by 56%. 

Slightly different patterns on the association between religious participation and cognitive impairment were found for oldest-old men (see Table 3). For men, religious participation reduced the odds of being cognitive impaired by a substantial factor of.35 (*P* < .001). Tests of mediating effects of positive psychological feelings suggest that male respondents, who were optimistic and happy, exhibited significant lower odds of being cognitive-impaired. By adding leisure activities into Model 3, both the coefficient and significance level of religious participation reduced a bit, suggesting that playing cards and/or mah-jong, and watching TV and/or listening to the radio played significant role in mediating the effects of religious participation. Among oldest-old men, no significant urban/rural differences in cognitive impairment were found. The effects of being married were particularly substantial and significant for oldest-old men. 

In sum, religious participation was found to be significantly associated with lower odds of cognitive impairment for both sexes, but the association was a little stronger for men than for women. Positive psychological feelings and the majority of leisure activities partially mediated the association between religious participation and cognitive impairment for both gender groups.

## 5. Discussion

Although there is some evidence suggesting that religious involvement is associated with better cognitive aging in the U.S., there have been no analyses of such relationship among Asian population living in Confucian society. In addition, little is known about the potential indirect effects of religious involvement [9] and how the effects might condition on demographic factors such as gender. This study examined how cognitive impairment is associated with religious participation, control, mediating, and moderating factors among the oldest-old Chinese. The major findings of this study include: (1) levels of religious participation are negatively and significantly correlated with cognitive impairment among the oldest-old Chinese; (2) positive psychological feelings and leisure activities engagement partially explain the association; (3) there are gender differences in levels of religious participation as well as in the relationship between religious participation and cognitive impairment: even though oldest-old women report more religious participation than oldest-old men on average, it is the latter who are likely to gain more cognitive benefits from religious participation. 

Results of this study suggested that participation in religious activities is significantly associated with cognitive functioning among older Chinese. In China, different religious traditions tend to coexist peacefully and each of them serves distinctive cognitive benefits: confucianism provides life philosophies and ethical teachings to establish proper relationships between persons and develop individual moral nature (e.g., [42, 43]); Taoism encourages harmony and balance to underlie the mind-body connection (e.g., [16]); ancestor worship bridges the dead with the living to provide psychological comfort; Buddhism brings optimisms and hope to promote spiritual well-being (e.g., [15]). Collectively, it can be speculated that religious participation in China, a unique form of social engagement as well as lifestyle, may be directly and indirectly related to cognitive functioning over and above controls for demographics, ADLs, and psychosocial mediators. 

This study examined two sets of psychosocial mediators. Participating in various religious activities is significantly associated with optimism, hope, and happiness and an engaged lifestyle, all of which are beneficial for cognitive functioning. For instance, playing cards or Mah-jong is intellectually challenging, requiring sophisticated strategies, calculations, and collaborations. These games are very popular among older Chinese, especially among coreligionists who get to know each other through religious involvement. Although previous studies in the U S present evidence that watching TV could lead to distress because it increases fear, mistrust, and alienation [44, 45], watching TV was found to be negatively correlated with psychological distress among older Chinese [19]. This indicates that TV programs in China may be more intellectually engaging than those in the U.S. because they show more informative programs, history programs, and operas and fewer situation comedies. Older Chinese with few other sources of information may find TV watching more intellectually challenging and informative. In addition, watching TV in China is probably also more socially integrated than in the U.S. because fewer televisions and larger households mean more people will watch TV and discuss the content of TV programs together. 

Differential involvement in religious participation as well as differential impact of religious participation on cognitive impairment is identified in this study. Consistent with some of the previous studies on religion and health, women are more involved in religious activities on average than men (e.g., [29, 46, 47]), but men tend to enjoy more cognitive benefits. These findings suggest the potential gender differences in kinds of religious participation: While oldest-old Chinese women, the majority of whom are illiterate, may engage more in ritual activities such as temple visiting and collective prayer, which might be beneficial for their longevity [17], the better educated oldest-old men may engage more in other kinds of religious activities such as scriptural discussion, event organization, and philosophical thinking that are more intellectually stimulating and have direct cognitive benefits. In other words, the kinds of religious activities in which oldest-old Chinese men are engaging may amplify their pre-existing cognitive advantages. 

The results of this investigation are intriguing; however, much remains to be explored. The first priority is the measurement improvement. Future research should examine the content of religion and spirituality practice or beliefs rather than treating them as social or demographic components such as simple measures of education and income. The measure of religious participation needs to be further decomposed to identify the precise facet of religion that is responsible for cognitive functioning. Does it solely reflect public participation, similar to organizational participation in the U.S., or reflect private religious practice such as praying at home or life philosophies such as Confucianism and Taoism? Although the follow-up in-depth interviews in China suggested that religious participation is likely to be multidimensional, more well-developed questions gauging multiple facets of religiosity including religious affiliation and life history dimensions of religious involvement need to be covered in future surveys so that their different effects on cognitive functioning can be precisely evaluated empirically. Also, with more sophisticated measures of religiosity and mediators and more advanced study design in the future, we will be able to find out whether religious participation is simply an ordinary form of activity that has neuroprotective functioning or it has its unique contribution in mobilizing cognitive faculties. 

In addition, measures of social support, which are: (1) central to religion; (2) found in diverse religious settings; (3) related empirically with health (e.g., [46]), need to be identified and included in future surveys such that the effects of religion-based social support on cognition can be tested in comparison with other types of social support from nonreligious settings. Besides, given the possibility of a reciprocal relationship between religious participation and cognitive impairment, longitudinal data covering longer period of time is essential in examining the causal effects of religious involvement on the change status of cognitive functioning over time. More comprehensive samples covering younger generations are likewise imperative. One of the latest surveys on religious beliefs in China disclosed an astonishing recent trend in contemporary China. Approximately one fourth of the Chinese population or more than 300 million Chinese, are involved in religious activities of one form or another. Among the 1,435 religious believers surveyed, approximately 62 percent are in the 16–39 age group while only 9.6 percent are 55 years old or older [48]. This is in sharp contrast to the fact in the end of last century when senior believers formed the dominant segment of religious group. Although it is not clear whether current younger adults are more likely to identify themselves as religious, it will be critical to include them in future surveys to examine age variations in religious participation, perception of meanings of religion, as well as the effects of religion on health.

## 6. Conclusion

Findings of this study provide evidence suggesting the positive association between religious participation in China and cognitive functioning among the oldest-old. Much of the association between religious participation and cognitive impairment is accounted for by the proposed psychosocial mediators. In terms of gender differences, women reported higher proportion of religious participation, but the beneficial effects of religious participation on cognitive functioning were stronger for men than for women. Findings indicate that (a) religious participation is significantly correlated with cognitive functioning in part because religious oldest-old are more likely to be optimistic and happy and engage in more cognitively stimulating, socially integrated, and physical activities; (b) there might be gender differences in religious participation such that oldest-old men may engage in kinds of religious activities that are particularly relevant to cognitive functioning.

## Figures and Tables

**Table 1:** Characteristics of the Oldest-Old in China, by Gender: 1998.

Variable	Male *n* = 3, 516	Female *n* = 5, 187
Demographics		
Age (years)	89.9	93.4
Literate (%)	62.9	12.7
Urban residence (%)	40.0	36.2
Married (%)	33.2	5.0
Han (%)	93.3	92.7
Number of ADL disabilities (0–6)	0.7	1.3
Religious participation (%)	11.1	19.7
Positive psychological feelings		
Optimism (%)	77.1	65.8
Happiness (%)	49.7	40.5
Leisure activities		
Exercise (%)	38.6	19.3
Garden work, grow vegetables (%)	12.6	5.2
Reading newspaper or books (%)	32.7	5.6
Playing cards and/or mah-jong (%)	17.2	9.2
Watching TV and/or listening to radio (%)	62.5	43.8
Cognitive impairment (%)	13.8	34.0

Notes: (a) ADL = activity of daily living; (b) *N* = 8,703.

**Table 2:** Estimated Odds Ratios for religious participation, demographics, and mediators, on cognitive impairment for Women (Logistic Regression Estimate: Oldest-Old Chinese 1998).

Variable	Model 1	Model 2	Model 3
Religious participation	.56***	.62***	.70**
	(–.58)	(–.47)	(–.36)
Positive psychological feelings			
Optimism (%)		.31***	.32***
		(–1.18)	(–1.14)
Happiness (%)		.50***	.54***
		(–.69)	(–.61)
Leisure activities			
Exercise (%)			.70***
			(–.36)
Garden work, grow vegetables (%)			.71
			(–.34)
Reading newspaper or books (%)			.95
			(–.05)
Playing cards and/or mah-jong (%)			.34***
			(–1.08)
Watching TV and/or Listening to radio			.63***
			(–.46)
Number of ADL disabilities (0–6)	1.56***	1.49***	1.44***
	(.44)	(.40)	(.37)
Demographics			
Age (years)	1.11***	1.11***	1.10***
	(.10)	(.11)	(.10)
Literate (%)	.49***	.49***	.56***
	(–.72)	(–.72)	(–.58)
Urban residence (%)	.72***	.80**	.92
	(–.33)	(–.22)	(–.09)
Married (%)	.56*	.55*	.57*
	(–.58)	(–.59)	(–.59)
Han (%)	.99	1.21	1.32*
	(–.01)	(.19)	(.28)
–2 Log Likelihood	4935.77	4515.80	4424.91
*χ* ^2^/d.f.	1712.23/7	2132.20/9	2223.09/14

Notes: (a) ADL = activity of daily living; (b) *N* = 5,187; (c) *P < .05; **P < .01; ***P < .001; (d) Two-tailed significance test is used; (e) Unstandardized logistic regression coefficients are presented in parentheses.

**Table 3:** Estimated Odds Ratios for religious participation, demographics, and mediators, on cognitive impairment for Men (Logistic Regression Estimate: Oldest-Old Chinese 1998).

Variable	Model 1	Model 2	Model 3
Religious participation	.35***	.39***	.46**
	(–1.06)	(–.95)	(–.79)
Psychological status			
Optimism (%)		.27***	.37***
		(–1.33)	(–.99)
Happiness (%)		.37***	.41***
		(–.99)	(–.89)
Leisure activities			
Exercise (%)			.75^+^
			(–.29)
Garden work, grow vegetables (%)			.84
			(–.18)
Reading newspaper or books (%)			.81
			(–.21)
Playing cards and/or mah-jong (%)			.61*
			(–.49)
Watching TV and/or Listening to radio			.45***
			(–.79)
Number of ADL disabilities (0–6)	1.73***	1.64***	1.57***
	(.55)	(.50)	(.45)
Demographics			
Age (years)	1.09***	1.10***	1.09***
	(.08)	(.09)	(.08)
Literate (%)	.55***	.60***	.74*
	(–.60)	(–.51)	(–.31)
Urban residence (%)	.80^+^	.98	1.23
	(–.22)	(−.02)	(.21)
Married (%)	.57***	.57***	.59***
	(–.57)	(–.57)	(–.54)
Han (%)	.73	.86	.99
	(–.32)	(–.15)	(−.01)
–2 Log Likelihood	2088.44	1860.94	1800.54
*χ* ^2^/d.f.	736.51/7	964.02/9	1024.42/14

Notes: (a) ADL = activity of daily living; (b) *N* = 3,516; (c) *P < .05; **P < .01; ***P < .001; (d) Two-tailed significance test is used; (e) Unstandardized logistic regression coefficients are presented in parentheses.
